# Supplementary material for: A systematic review: Role of dietary supplements on markers of exercise-associated gut damage and permeability
Source: PLoS One. 2022 Apr 13;17(4):e0266379. doi: 10.1371/journal.pone.0266379 (PMC9007357; doi:10.1371/journal.pone.0266379)
Supplement: S1 File — (DOCX) [file pone.0266379.s001.docx]

S1: Search strategy: A systematic review: role of dietary supplements on markers of exercise-associated gut damage and permeability

**PubMed**

(("gut"[tiab] OR gastrointestinal[tiab] OR GI[tiab] OR "intestines"[MeSH Terms] OR "intestinal"[tiab] OR "mucosal"[tiab] OR splanchnic[tiab] OR "microbiota"[MeSH Terms] OR "microbiota"[tiab] OR "microbiome"[tiab] OR "gastrointestinal microbiome"[MeSH Terms])

AND ("permeability"[MeSH Terms] OR "permeability"[tiab] OR leaky[tiab] OR hyperpermeability[tiab] OR "function"[ tiab] OR "dysfunction"[ tiab] OR "injury"[ tiab])) AND ("exercise"[MeSH Terms] OR "exercise"[tiab] OR "training"[ tiab] OR endurance[tiab] OR "physical activity"[tiab]) NOT("animals"[MeSH Terms] NOT "humans"[MeSH Terms])

AND “dietary supplements”[MeSH Terms] OR “dietary supplements”[tiab] AND “functional food”[MeSH Terms] OR “functional food”[tiab] AND “probiotic”[tiab] OR “probiotic”[MeSH Terms]

**Cochrane**

#1. MeSH descriptor: [Intestines] explode all trees

#2. MeSH descriptor: [Microbiota] explode all trees

#3. ("gut" or "gastrointestinal" or "GI" or "intestines" or "intestinal" or "mucosal" or "splanchnic" or “microbiota” or “microbiome”):ti,ab,kw

#4. ("Permeability" or "leaky" or "hyperpermeability" or "function" or "dysfunction" or "injury"):ti,ab,kw

#5. ("Exercise" or "training" or "endurance" or "physical activity"):ti,ab,kw

#6. MeSH descriptor: [Animals] explode all trees

#7. MeSH descriptor: [Humans] explode all trees

#8. ((#1 or #2 or #3) and #4 and #5) not (#4 not #5)

**Ebsco (CINAHL, MEDLINE, SPORTDiscus)**

S1. (MH “intestines”)

S2. (MH “Gastrointestinal Microbiome”)

S3. (MH “Microbiota”)

S4. (TI "gut" OR AB “gut”) OR (TI “gastrointestinal” OR AB “gastrointestinal”) OR (TI “GI” OR AB “GI”) OR (TI "intestinal" OR AB "intestinal") OR (TI “intestines” OR AB “intestines”) OR (TI "mucosal" OR AB “mucosal”) OR (TI “splanchnic” OR AB “splanchnic”) OR (TI “microbiota” OR AB” microbiota”) OR (TI “microbiome” OR AB” microbiome”)

S5. (MH "permeability")

S6. (TI "permeability" OR AB "permeability") OR (TI “leaky” AB “leaky”) OR (TI “hyperpermeability” OR AB “hyperpermeability”) OR (TI "function" OR AB “function”) OR (TI "dysfunction" OR AB “dysfunction”) OR (TI "injury" OR AB “injury”)

S7. (MH “exercise”)

S8. (TI "exercise" OR AB “exercise”) OR (TI "training" OR AB “training”) OR (TI “endurance” OR AB “endurance”) OR (TI "physical activity" OR AB “physical activity”)

S9. (MH "animals") NOT (MH "humans")

S10. (S1 OR S2 OR S3 OR S4) AND (S5 OR S6) AND (S7 OR S8) NOT S9
