# Supplementary material for: A systematic review: Role of dietary supplements on markers of exercise-associated gut damage and permeability
Source: PLoS One. 2022 Apr 13;17(4):e0266379. doi: 10.1371/journal.pone.0266379 (PMC9007357; doi:10.1371/journal.pone.0266379)
Supplement: S2 File — (DOCX) [file pone.0266379.s002.docx]

S2: Summary of the risk of bias for individual studies

Paper: **A SYSTEMATIC REVIEW: ROLE OF DIETARY SUPPLEMENTS ON MARKERS OF EXERCISE-ASSOCIATED GUT DAMAGE AND PERMEABILITY**

Authors: Sarah Chantler, Alex Griffiths, Jamie Matu, Glen Davison, Adrian Holliday, Ben Jones

Contact details: Sarah Chantler, [s.a.chantler@leedsbeckett.ac.uk](mailto:s.a.chantler@leedsbeckett.ac.uk)

Table 1: Summary of the risk of bias for individual studies

|  | **Selection bias** | | **Performance bias** | **Detection bias** | **Attrition bias** | **Reporting bias** | **Other bias** |
| --- | --- | --- | --- | --- | --- | --- | --- |
|  | Sequence generation | Allocation concealment | Blinding of participants / personal | Blinding of outcomes | Outcome data | Selective reporting |  |
| Axelrod et al (2019) | 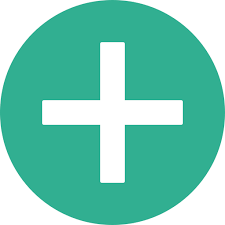 | 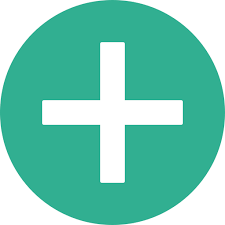 | 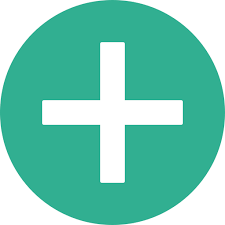 | 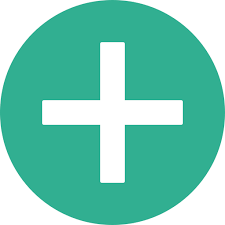 | 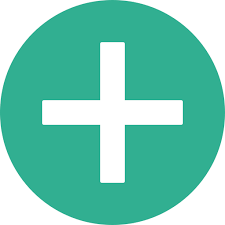 | 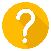 | 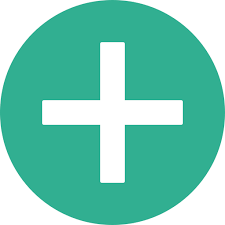 |
| Buchman et al (1999a) | 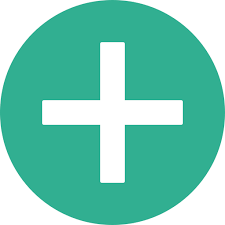 | 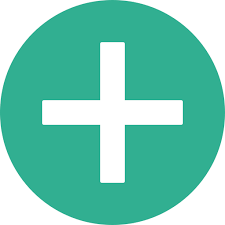 | 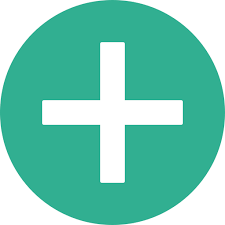 | 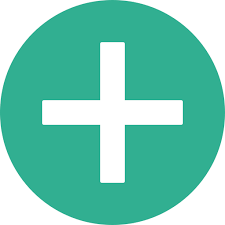 | 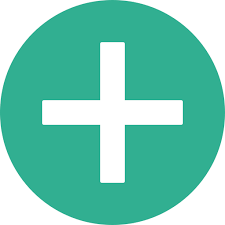 | 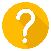 | 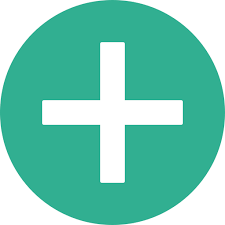 |
| Buchman et al (1999b) | 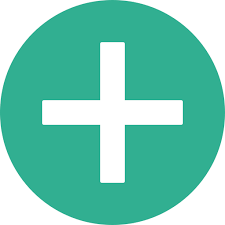 | 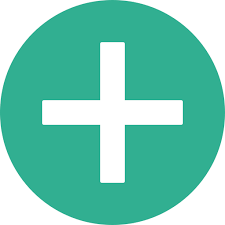 | 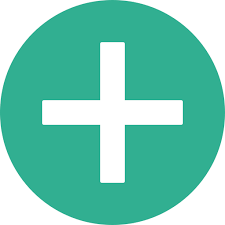 | 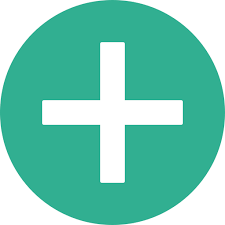 | 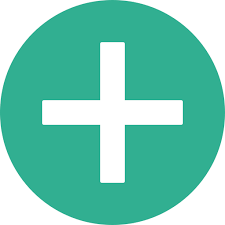 | 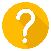 | 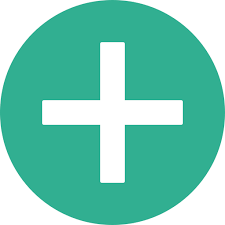 |
| Costa et al (2019) | 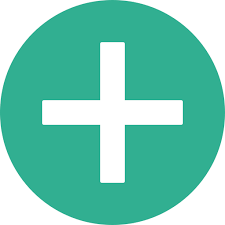 | 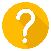 | 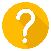 | 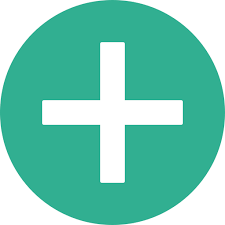 | 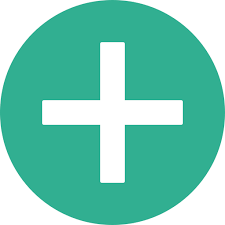 | 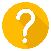 | 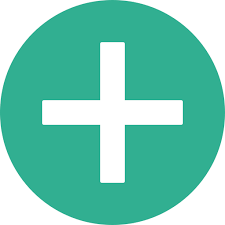 |
| Davison et al (2016) | 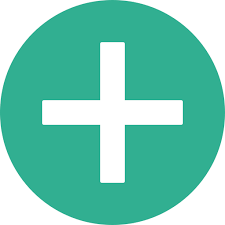 | 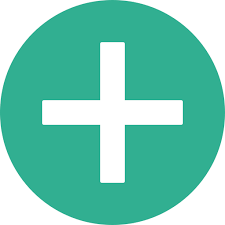 | 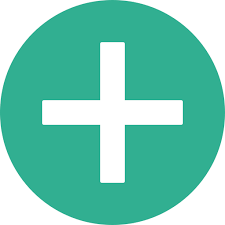 | 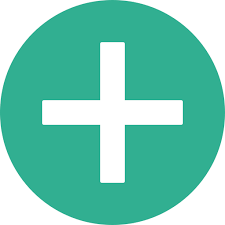 | 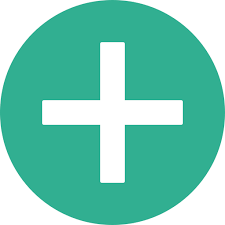 | 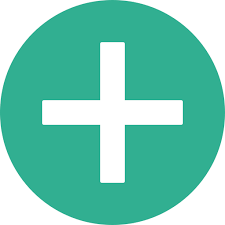 | 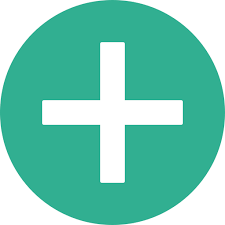 |
| Flood et al (2020) | 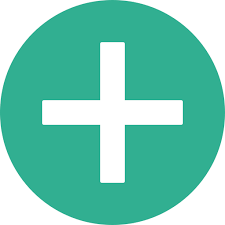 | 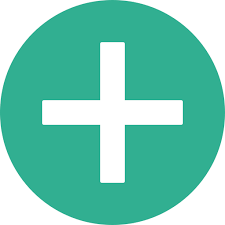 | 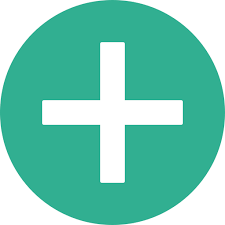 | 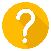 | 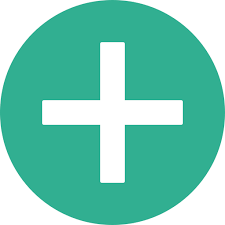 | 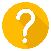 | 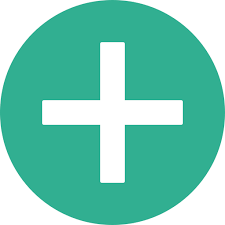 |
| Jonvik et al (2019) | 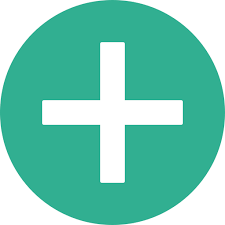 | 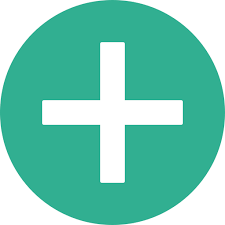 | 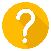 | 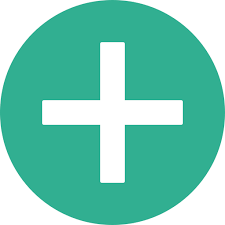 | 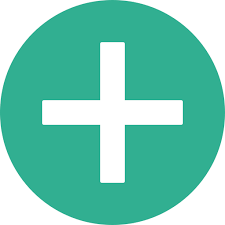 | 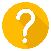 | 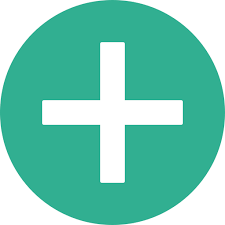 |
| Karl et al (2017) | 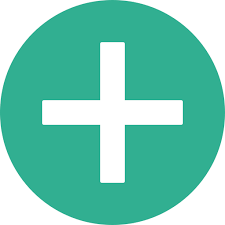 | 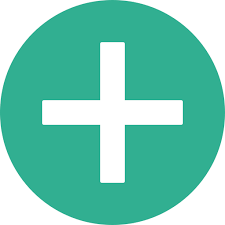 | 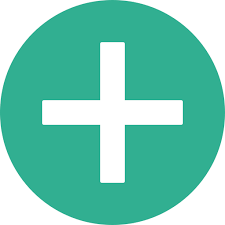 | 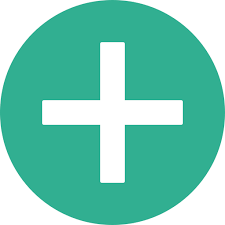 | 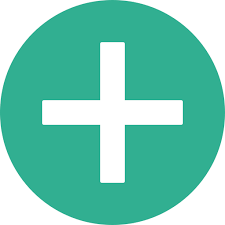 | 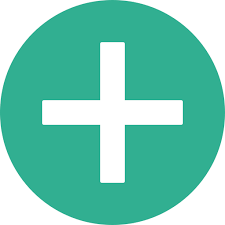 | 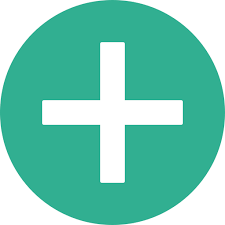 |
| Kartaram et al (2019) | 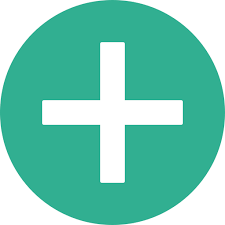 | 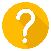 | 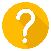 | 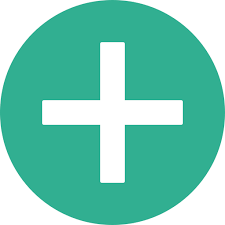 | 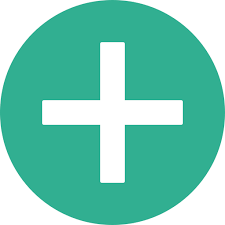 | 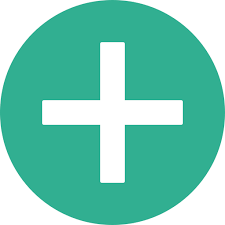 | 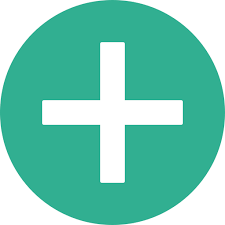 |
| Lambert et al (2008) | 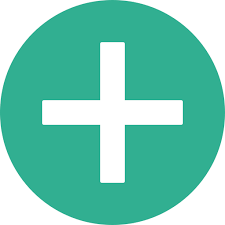 | 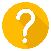 | 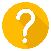 | 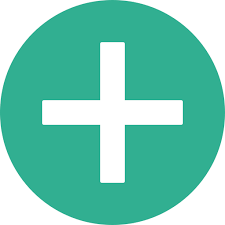 | 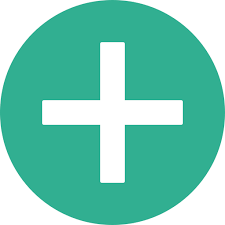 | 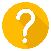 | 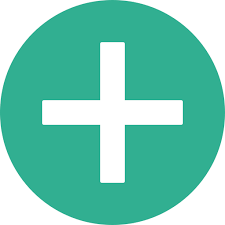 |
| Ma et al (2020) | 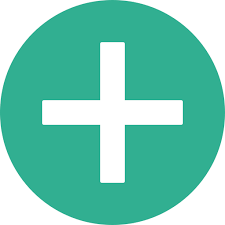 | 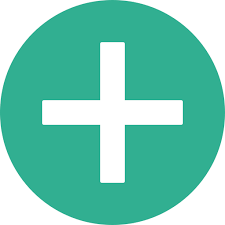 | 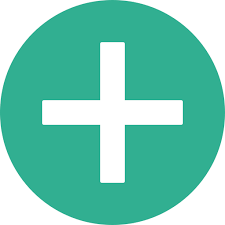 | 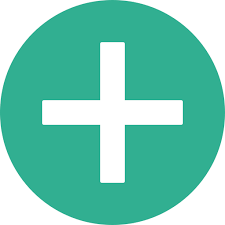 | 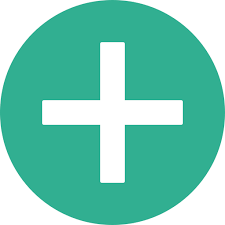 | 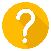 | 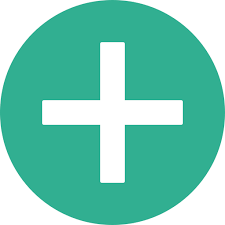 |
| March et al (2017) | 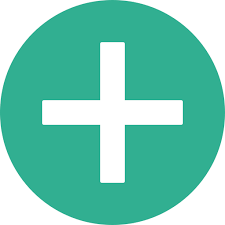 | 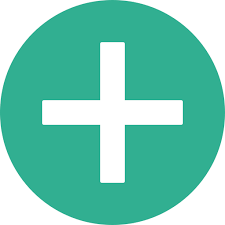 | 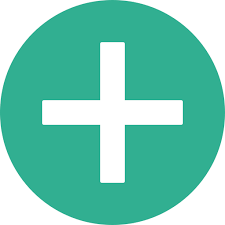 | 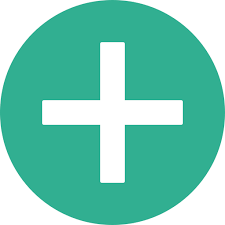 | 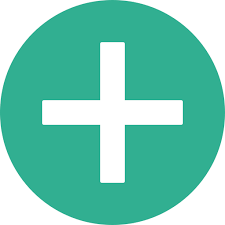 | 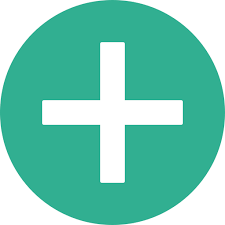 | 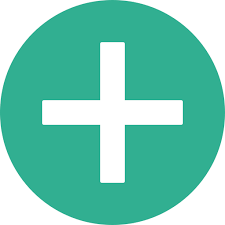 |
| March et al (2019) | 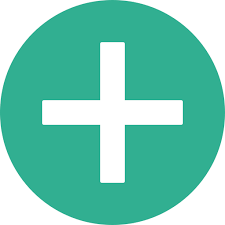 | 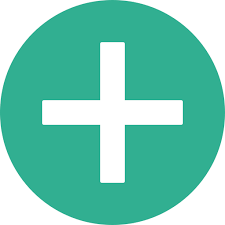 | 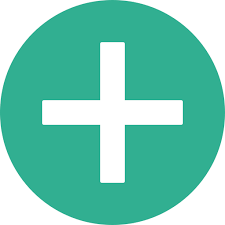 | 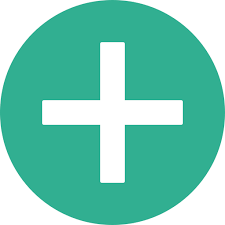 | 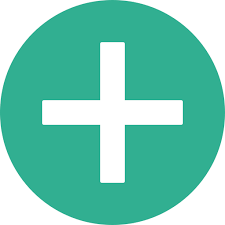 | 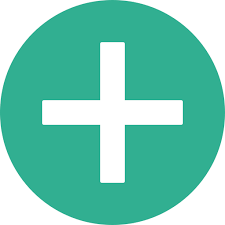 | 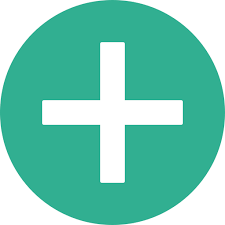 |
| Marchbank et al (2011) | 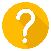 | 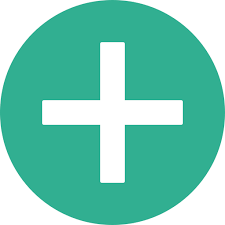 | 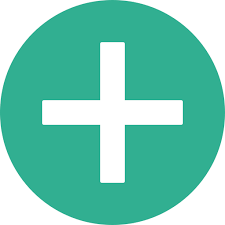 | 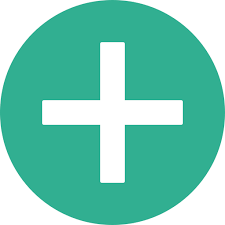 | 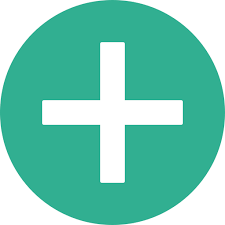 | 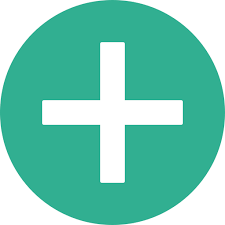 | 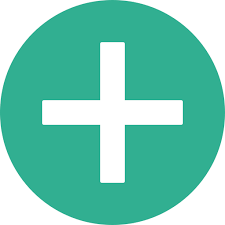 |
| McKenna et al (2017) | 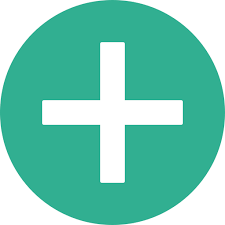 | 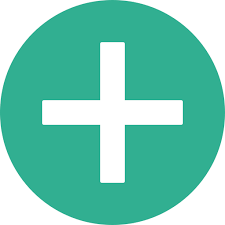 |  |  |  |  |  |
| Mooren et al (2020) |  |  |  |  |  |  |  |
| Morrison et al (2014) |  |  |  |  |  |  |  |
| Osborne et al (2019) |  |  |  |  |  |  |  |
| Pugh et al (2017) |  |  |  |  |  |  |  |
| Pugh et al (2019) |  |  |  |  |  |  |  |
| Pugh et al (2020) |  |  |  |  |  |  |  |
| Sessions et al (2016) |  |  |  |  |  |  |  |
| Snipe et al et al (2017) |  |  |  |  |  |  |  |
| Szymanski et al (2018) |  |  |  |  |  |  |  |
| van Wijck et al (2014) |  |  |  |  |  |  |  |
| Zuhl et al (2014) |  |  |  |  |  |  |  |
| Zuhl et al (2015) |  |  |  |  |  |  |  |

, low risk; , uncertain risk, , high risk
