# Supplementary material for: A systematic review: Role of dietary supplements on markers of exercise-associated gut damage and permeability
Source: PLoS One. 2022 Apr 13;17(4):e0266379. doi: 10.1371/journal.pone.0266379 (PMC9007357; doi:10.1371/journal.pone.0266379)
Supplement: Attachment — Submitted filename: Response to reviewers PLOS one dec 2021.docx [file pone.0266379.s005.docx]

School of Sport

Leeds Beckett University

Leeds

LS6 3QS

Telephone: +44 (0) 1138122170

E-mail: [s.a.chantler@leedsbeckett.ac.uk](mailto:s.a.chantler@leedsbeckett.ac.uk)

Dear Krzysztof Durkalec-Michalski and editorial team,

**RE: Response to reviewers**

Thank you for giving us the opportunity to revise our manuscript again. We would like to thank the reviewers for their suggestions and comments, which we feel have enhanced the quality and accuracy of the manuscript.

We have responded to all the comments and suggestions individually below. All modifications in the manuscript have been made using track-changes as requested. For clarity, the page numbers referred to in this reply are based on the track changes copy, and not the clean copy.

Thank you again for your time and assistance.

Best regards,

Sarah Chantler

On behalf of the authors

**Response to the reviewers:**

Thank you for taking the time to review the manuscript again. We have aimed to address your concerns below and hope you will find our changes to your satisfaction.

**Editor Comments**

Thank you for your input. I have added ‘amended’ to the comments that were changed without any need for further discussion.

1. Line 41 – it should be clearly indicated that authors mention reduction in “selected” markers. Amended
2. Conclusion, taking into account the large heterogeneous group of treatments (various supplements, euhydration, carbohydrates) should be formulated much more carefully.

Thank you, the conclusion of the abstract has been adjusted accordingly to reflect your comment:

Line 47: “In spite of a large heterogeneity across the selected studies, appropriate inclusion of different nutrition strategies could mitigate the initial phases of gastrointestinal cell disturbances in athletes associated with exercise.”

3. Line 66 and 128 - the consent number of the indicated bioethics committee is missing. Added
4. Line 87 – the double space should be removed. Amended
5. Line 89 - The double dot must be removed. Amended
6. Lines 248, 249 and 251 - standardize spacing (insert space). Amended
7. In tables, other co-authors should be respected and where applicable, "et al." should be written next to the first author's name. Amended
8. Line 91 - as you indicate that it is mean values - remove the unnecessary standard deviation. Amended
9. Line 111 - It should be pointed out that "nutritional support" rather than only "supplementation" is particularly important in the discussed topic. Amended
10. Line 138 – the authors wrote that “database can be found in supplementary material” – Please indicate clearly where exactly these data are.

Thank you, I have reformatted the indication for all supplementary materials as S1, S2, S3 as per the author guidelines.

11. Line 152 – “an increase in plasma concentration” – but “an increase” of what? Please clarify. Clarified
12. Please insert "i-FABP" for the first time when using "Intestinal fatty acid binding protein" for the first time. Amended
13. Line 165 – the authors wrote: “Supplements, by the IOC definition, can include any food” - Food is not the same as supplements. These concepts should not be confused.

This definition is directly attributed to the IOC position stand on supplementation. From the position stand:

“…we recognise that dietary supplements come in many forms, including the following:

1. functional foods, foods enriched with additional nutrients or components outside their typical nutrient composition (eg, mineral-fortified and vitamin-fortified, as well as nutrient- enriched foods)

2. formulated foods and sports foods, products providing energy and nutrients in a more convenient form than normal foods for general nutrition support (eg, liquid meal replacements) or for targeted use around exercise (eg, sports drinks, gels, bars)”

This definition therefore can include carbohydrate and protein, in both supplemental or sports food (carbohydrate gel) or food based form. Since a portion of the studies selected included nutrients in powder, capsule, sports food and food form – it was felt this definition would be appropriate (Karl et al, 2017). If the intention of the food or food derivative is to supplement the existing diet (e.g. around exercise) then it can be included in the definition. This gives clarity to the logic behind food changes (e.g. FODMAP or gluten) not being included in the review.

14. Line 160 – the space between “/” and “low” should be removed. Amended
15. Lines 182-185 - This section is convoluted and worth refuting. Besides, the authors write about "a number of studies" and cite one work.

Thank you for your comment. The sentence has been rewritten to try improve clarity, with the other studies that did not include a resting control. It now reads:

Line 191: “The studies that included DSAT in their methodology but had no resting control were excluded from data extraction for that marker (Flood et al., 2020; Pugh, J. N. et al., 2020; Snipe et al., 2017; Wijck, Van et al., 2014).”

16. Line e.g. 182 and where applicable - Please make sure that "dual saccharides" is the correct statement in this context and that it should not be "disaccharides". Thank you, I have written the full name of the test; dual saccharide absorption test.
17. Line 197 – the authors wrote: “In total, thirty studies met the inclusion criteria”. However, in the pointed figure 1 or in the line 230,  27 works are indicated. Please correct properly throughout the work truthfully. Amended
18. Lines 205-208 – second part of this sentence is quite difficult to follow. Please consider to clarity this.

Thank you for highlighting this. The sentence has been changed to read:

Line 206: “Data were extracted independently by two researchers (SC and AG) into a standardised spreadsheet, which included the characteristics of articles valid for review; including gastrointestinal damage and permeability markers.”

19. Lines 225-227 – the authors wrote "Based on previous work ..." - however, there are no citations in this regard. This should be completed. Amended
20. Lines 246-248 - The sentence is a bit vague - please clarify. it is a bit incomprehensible what this temperature is about - is it exercises at this temperature?

To improve the reading, the sentence now reads:

Line 251: “Dosage-matched (20g·day-1) studies evaluating endothelial damage (i-FABP) showed a positive blunting effect of colostrum subsequent to exercising at 22 ˚C (March et al., 2017) and 30˚C (March et al., 2019) but not at 40˚C (McKenna et al., 2017).”

21. The notation of units is not uniform at work, e.g. in line 251 the authors write "> 120g.day^-1^) (39)" and in line 246 "(20g · day^-1^") - it should be standardized (here it is about "dot"). Changed
22. The tables require very thorough corrections. They are not uniform when it comes to editing and description. In some sections no clear marker is indicated, intervals are not uniform, no indication of what was given as placebo, gaps of necessary units for some markers, spacing, missing or incomplete parentheses, no indication of "et al." after the first authors, it is not clear in some records whether powder or capsules were administered, the significance value is missing in some records, some units due to strange notation unclear, the accuracies of the reported data within some records should also be standardized. Furthermore, strains of probiotics should be clearly stated.

Thank you for highlighting the errors and inconsistencies. We have made every effort to adjust the table to reflect the data extracted from the studies in the most consistent way possible. Units, spacing, authors (et al), placebos, significance values have been checked and aligned.

23. On page 12 (Table 1) of Pugh et al. (2017), the authors misinterpreted the amount of glutamine (09g…). Do I understand correctly that it should be 0.9 g…? Amended
24. On page 12 (Table 1) - unit for correction in Osborne et al. (2019). Now it is: "ƞg·ml^-1^". Amended
25. Unclear sign (page 13, table 1, Mooren et al. (2020): "<" before the age of the respondents ("18-35 years"). Amended
26. What does "placebo diet" mean? (page 14, table 1). In addition, in this record also probably lacks the results of one group (Karl et al., (2017).

Thank you. The table has been adjusted to reflect the placebo condition as the standard diet (14.6 MJ/~3500kcal·day) and to show the three groups in the findings more clearly.

27. With some records, the work number (from the references) is not indicated (e.g. with "Ma et al. (2020)"). Amended
28. Additionally, in the "Ma et al. (2020)" record, there is an error in the writing of markers - writing and using "/" suggests that it is "i-FABP / Creatine ratio". Besides, the authors spelled it wrong - there shouldn't be "Creatine" but "Creatinine". The above example shows that all markers in the table and the work should be carefully checked by the Authors for serious mistakes in this respect. Thank you for highlighting this error. It has been amended.
29. Table 1, page 15 (Kartaram et al. (2018) and line 327 - what do the authors mean "ad libertum"? Shouldn't it be "ad libitum"? Amended
30. Line 272 – correct dose - shouldn't it be like in the table (0.25g…)? Amended
31. Line 274 - why "likely" is in italics? The paper uses magnitude-based inferences as their statistical model, and use *likely* in italics to differentiate from standard interpretation. To improve the understanding, it now reads:

Line 280: “Magnitude based inferences suggested a *likely* lower i-FABP levels post-exercise in the 0.5 and 0.9g·kg^-1^ FFM conditions compared to the placebo (Pugh, J. et al., 2017).”

32. Line 279 – correct unit. Amended
33. Line 402 - some blue colour is inserted. Amended
34. Line 407 and 412 – remove the double dot. Amended
35. Lines 414-419 - The topic of Colostrum and doping issues and WADA is a repeated false myth. You should clearly state your factual position on this matter and not repeat these myths.

Thank you for your concern around this topic. The authors have aimed not to misrepresent any of the existing data on this topic. However, for practitioners working in sport; it is also important to acknowledge that this is still on the WADA website: <https://www.wada-ama.org/en/questions-answers/prohibited-list-qa#item-388> Question 8. All risk management for athletes should have all the available information. We have removed the first sentence to allow for less possible confusion on this topic.

36. The section on "Glutamine" - the work in a way recommends the effectiveness and use of GLU. However, the reader should clearly indicate the potential risk and the negative one, especially related to the supply of high doses. It has been written extensively about this in the literature.

Thank you for the suggestion. Recent literature has showed that dosages up to 0.9g·kg/day are tolerated at rest (Ogden et al., 2020) and there were no issues reported in the study by Pugh et al (2017) at the higher dosages. We have included an additional sentence to highlight this possible concern:

Line 463: “Pugh et al (2017) illustrated a possible dose-dependent benefit but was the only study to compare different dosages. Higher dosages (0.9g·kg) had minimal impact on GIS in spite of some concerns around tolerance (Pugh, J. et al., 2017).“

We have included a further sentence around the justification on long term supplementation:

Line 490: “To note, chronic high dosages are not recommended due to concerns over long term effects (Holecek, 2013).”

37. Line 445 – change “…by Zuhl and colleagues (43,44) This may highlight” to “…by Zuhl and colleagues (43,44), and may highlight”. Amended
38. Line 489 – use the impersonal form in this sentence. Adjusted
39. Line 495 - insert the number of the cited work. Amended
40. Lines 515-516 - I do not agree with the authors on this point, because there has been some work in this regard - they should be reached.

We are unaware of any research in humans that will illustrate the energy expenditure of the gastrointestinal lining in healthy participants, or around exercise and endothelial recovery. If the editor is aware of research in this area, we would greatly appreciate sharing. Due to concerns over inaccuracy, the text has been adjusted:

Line 560:” These gastrointestinal associated energy costs may be higher in athletes over longer duration exercise.”

41. Line 546 - "creatine" or "creatinine" levels? Changed
42. Line 563 – correct the abbreviation. Amended
43. In the conclusions you generally write about some specific "carbohydrate" or should be "carbohydrates"? Amended
44. Notation of some references should be corrected. Amended
45. The surname "de Oliveira" should be spelled "de Oliveira" not "Oliveira de". Should be corrected in the literature record and where applicable. Apologies but I am unsure where this is in the reference list, as I have not referenced their work.

**Comments to the Author** **from Reviewer #3**

Thank you for your comments and suggestions to improve the content of our paper.

Reviewer #3: Overall:
Reviewer #2: The authors provide a systematic review of a variety of dietary supplements and their impact on exercise-associated gut damage and gut permeability. The topic is highly interesting. A few suggestions are provided by section below.
Nevertheless, all the chosen supplements were just briefly explained in this paper. The results are mainly in the table. There are no characteristics of the chosen substances, nor the mechanisms underlying their effects on gastrointestinal problems during exercise, nor the explanation why these substances where chosen for this review (except the fact that there are studies using this supplements for GI tract problems). In my opinion there are too many, not related to each other (for example fluid restrictions in the same review as glutamine), kinds of supplements investigated just briefly in this review.

Thank you for your comments. The authors agree that the review did cover multiple supplements, but that the choices are directed by the outcome measures. The choices were revealed by the search process rather than a more narrative review approach which could have been more selective around the mechanisms.

Introduction
Line 95-97: You mention just the increased immune response, not a state called immunodepression, which is even a more significant problem after a bout of acute exercise.

Thank you for the suggested revision. We have a concern that immunodepression is a more general term associated with the changes post-exercise, often localised around the upper respiratory tract, and general inflammatory cytokines (Moreira et al., 2007).

Due to the tenuous link between gastrointestinal cell damage and the expression of gastrointestinal symptoms, there is evidence for some immune response, but not necessarily immunodepression. We felt we did not want to over-estimate the immune response in this explanation, and therefore have not incorporated the suggested wording.

Line 97-98: What does a shorter duration exercise mean? In comparison to previously mentioned ultra-endurance and long distance.

Thank you, the sentence has been adapted to clarify the timing – <2-3 hours which may be considered shorter than the ultra-endurance events.

Methods
Line 132: I can see what keywords were used to perform the search, but nothing is mentioned about the exact index terms that were used.

Thank you, these are in the search terms included in the supplementary material (S1).

165-177: I don’t quite understand why this paragraph is in the Methods section rather than in the Introduction.

Thank you for your comment. When considering the variety of research available, it was important to differentiate between different inclusion and exclusion criteria. Therefore, the definition applied to the inclusion criteria would be more suitable in the methods rather than the introduction.

Line 181: Was the type of placebo used in the research important for this Review outcomes?

Thank you for the question. We have included the content of the placebo substances into the table. When appropriate, the authors aimed to include this in the discussion around the limitations.

Line 188-189: Why there were no restrictions placed on the training status of participants?
Thank you for the question. We have elaborated in Line 196: “No restrictions were placed on the training status or sex of the participants as current studies available do not indicate there are any sex-based differences or training effect (Morrison et al., 2014; Snipe & Costa, 2018). “

Morrison et al (2014) found no difference in the trained and untrained groups in response to supplementation; and Snipe et al found no significant differences in the response to exercise between the sexes. These limited studies do not give sufficient grounds to refine the inclusion criteria further.

Results:
Line 234-235: “Nineteen studies (70%) of the studies used..” – there is something wrong with the style.

Thank you, this has been corrected.

Line 246: You didn’t mention whether bovine colostrum dosages where acute or chronic.

Thank you. These details can be found in Table 1. We have included an additional sentence to assist with clarity:

Line 256: “Studies ranged from 2 to 14 days.”

Further, there is a summary of the acute vs chronic protocols in the results that may be useful.

Line 241: “Supplementation protocols ranged from a single dose pre and/or during exercise (n=12, 24 hours pre-exercise) to multiple days of daily supplementation (n=15; up to 8 weeks).”

Line 251: Why did you mention that the dosage in this study was > 120 g/day? It was 1,7 g/kg/day and the average body mass was 75 kg in trained participants.

We felt that due to the other studies all having a dosage in g·day^-1^ that it may be useful to understand the estimated comparison. As this may have been estimated incorrectly from the authors, we have removed it.

What is more important about the colostrum supplementation studies is the placebo that is chosen. You didn’t mention it at all. It can make a huge difference when analysing the results of the supplementation vs placebo.

Thank you for highlighting this – we have included all the placebos used across the studies in Table 1.

332-341: Why is this paragraph placed right after the results of hydration status? It is quite odd for me and makes the reading of this review difficult.

Thank you for your comment. We have moved the reporting of risk of bias earlier in the results to prevent any confusion. (Line 254)

Discussion
361-363: I would expect thorough explanation of the mechanisms of colostrum on the gastrointestinal functions, not only mentioning and briefly describing that there are in vitro and in vivo studies.
Again there is nothing mentioned about the placebo used in the studies with colostrum supplementation.

Thank you for your comment. We have aimed to reference other previous reviews where the mechanisms are covered in more detail. The authors aimed to balance the content provided on each supplement. However, we understand that Bovine Colostrum has a much more extensive literature base to work from. We felt that the variability in the colostrum content itself based on milking times post-partum would be contribute more to results that the specific placebo used.

408-409: “therefore, it is unknown if training status alters the gut perfusion-exercise relationship” – are you confident enough to put this statement?

Thank you for your comment. Due to the single study, and limited research in this area, we have altered the wording to reflect something less categorical:

Line 430: “All, but one study (Mooren et al., 2020), used trained participants, and therefore, there is limited understanding if training status alters the gut perfusion-exercise relationship.”

410: Are you sure about the dosage?

Due to your concerns over the use of the mean weight reported in the study, we have removed this.

414-419: In my opinion, based on the previous research by Jones et al. you should reconsider putting this statement in this review, because it mas be understood that colostrum is a banned substance that shouldn’t be supplemented, which is completely untrue.

Thank you for your opinion. We have removed the first sentence of the paragraph to limit any confusion on our position of the current status. However, the wording from WADA is still important:

The wording from WADA: <https://www.wada-ama.org/en/questions-answers/prohibited-list-qa#item-388> : Colostrum is not specifically prohibited, however it contains certain quantities of IGF-1 and other growth factors which are prohibited and may influence the outcome of anti-doping tests. Therefore, WADA does not recommend the ingestion of this product.

For any practitioners or athletes, this is still to ensure that there is full transparency on the risk minimisation recommendations by WADA.

437-438 and 513-514: There is suddenly an animal based study mentioned, again just briefly, and not in the context of every supplement you mentioned in this Review.

Thank you for your comment. We have taken your comment under advisement to limit any reference to animal studies where possible and selected only studies done in human subjects.

458: I am convinced that nowadays we don’t use the term bacterial flora, instead you should write microbiota, in my opinion. Amended to reflect your suggestion.
