# Supplementary material for: A systematic review: Role of dietary supplements on markers of exercise-associated gut damage and permeability
Source: PLoS One. 2022 Apr 13;17(4):e0266379. doi: 10.1371/journal.pone.0266379 (PMC9007357; doi:10.1371/journal.pone.0266379)
Supplement: Attachment — Submitted filename: Response to reviewers PLOS one Feb 2022.docx [file pone.0266379.s006.docx]

**Response to reviewers:**

Please see our responses below to the most recent comments from the Editor

**Additional Editor Comments:**

1. **Line 190 - When using the term dual saccharides absorption test for the first time, put the abbreviation DSAT immediately - currently the reader may not know what this abbreviation is about.**

**Thank you for highlighting this, it has been changed.**

2. **Lines 453-458 and Editor’s remarks no.35 - this section should be removed as it is not based on scientific facts. Follow current WADA 2022 position and quote it (update ref 70) - Colostrum is not mentioned on this document. I agree that the reader must be informed about all aspects, but in the case of the ingredient Colostrum itself, it is repeating myths. WADA neither recommends nor prohibits it. But likewise, WADA does not recommend the use of any other supplement.**

**This section has been removed as requested.**

3. **In the newly introduced changes, double spaces are present in several places.**

Thank you, we have made all efforts to correct for any spacing issues.
