# Supplementary material for: A systematic review: Role of dietary supplements on markers of exercise-associated gut damage and permeability
Source: PLoS One. 2022 Apr 13;17(4):e0266379. doi: 10.1371/journal.pone.0266379 (PMC9007357; doi:10.1371/journal.pone.0266379)
Supplement: Attachment — Submitted filename: Reviewer comments june 2021 PLOS one 16 July 2021.docx [file pone.0266379.s004.docx]

Sarah Chantler

School of Sport

Leeds Beckett University

Leeds

LS6 3QS

Telephone: +44 (0) 1138122170

E-mail: [s.a.chantler@leedsbeckett.ac.uk](mailto:s.a.chantler@leedsbeckett.ac.uk)

Dear Pedro Tauler and editorial team,

**RE: Response to reviewers**

Thank you for giving us the opportunity to revise our manuscript. We would like to thank the reviewers for their suggestions and comments, which we feel have enhanced the quality of the manuscript.

We have responded to all the comments and suggestions individually below. All modifications in the manuscript have been made using track-changes as requested. For clarity, the page numbers referred to in this reply are based on the track changes copy, and not the clean copy.

The editor highlighted that the authors have external affiliations, however, these did not contribute to any funding associated with this research and were not in any way part of the research process, eliminating any risk of conflict of interest. However, for full transparency, GD has received research funding and honorarium from PanTheryx Inc., who supply bovine colostrum products to U.S., European, and Asia-Pacific markets, and received funding from Neovite Colostrum UK in 2008, 2010, and 2013. These would have been part of the declared funding in relation to the studies involving Bovine Colostrum, previously published by GD. However, since this work forms part of a doctoral thesis, separate to this research, we feel that there is no conflict of interest.

Thank you again for your time and assistance.

Best regards,

Sarah Chantler

On behalf of the authors

**Response to the reviewers:**

Thank you for taking the time to review the manuscript. We have aimed to address your concerns below, and hope you will find our changes to your satisfaction.

Reviewer #1: Dear author,

Thanks for submitting your manuscript. See below for my comments.

**Overall comments:**

1: It is quite hard to claim an effect on such a multifactorial problem. There is no causal cause between GIS and specific gut damage markers.

Thank you for your comment. While we agree that endothelial disruption does not directly cause gastrointestinal symptoms, the proposed sequence of physiological changes laid out in by Van Wijck (2012) and then Costa et al (2017) in Exercise Associated Gastrointestinal syndrome does link the initial endothelial damage to changes in endotoxin levels (Costa et al., 2017; Wijck, van et al., 2012). Endotoxin levels are not consistently associated to gastrointestinal symptoms, but may be part of the cumulative pathology and still bares investigating. We have added some further discussion around this to the introduction (L77-94), which we hope further clarifies these points.

2: Methods of included studies can be described more in depth to increase clarity.

Thank you for highlighting this. We have aimed to include as many aspects as possible of the various methodology in table 1, including exercise and supplementation protocol, participant details, as well as the data derived from the key biochemical markers. In order to ensure readability of the manuscript, this information is not repeated in text in order to limit the length of the paper.

3: With high carbohydrate intakes we often see fructose-intolerances (or even fructose allergics) which also leads to GIS: how are you sure about excluding these individuals?

Thank you for highlighting this. Due to each study’s own selection criteria, the participants are included as healthy and without any pre-existing GI disorders. This may assist in selecting out those who may suffer from a fructose intolerance or known reaction during exercise.

It is worth discussing that the addition of fructose to an existing glucose or maltodextrin drink may allow for higher absolute carbohydrate absorption and lower residual carbohydrate in the large colon, creating less risk for GIS (Oliveira, de & Burini, 2014). This seems to agree with some of the data from Wilson (2015) where rates of GIS were likely lower in the runners consuming a glucose/fructose mix compared to glucose alone (Wilson & Ingraham, 2015). The study by Flood et al (2020) is the only study in the review known by the authors to include a maltodextrin/fructose mix that exceeds ~60g/hour, with the other studies generally being lower in concentration or volume (Sessions et al., 2016). This may results in the other studies being less likely to have malabsorption or fructose-related GIS. Lastly, when using trained participants, there is a possibility that the small intestine upregulates the fructose and glucose transport proteins, again reinforcing that there is less risk of fructose being the direct cause for an increase in GIS (Horowitz et al., 1996). Lastly, we aimed to be clear that we were not evaluating the impact of supplements on GIS, and that fructose as a saccharide is unlikely to react or have different impact compared to other saccharides at the endothelial level in the small intestine.

**Specific comments:**

L77-89: The pathophysiology is poorly described. Please take a look at papers like de Oliveira et al 2014 and Costa et al 2016 and rewrite this specific part.

Thank you for your comment. We have adjusted the explanation; it now reads:

**Line 77:** “(Wijck, van et al., 2011). Endothelial cell damage increases permeability via disruption and dysregulation of tight junction proteins (Davison et al., 2016; Dokladny et al., 2016). . Increased permeability has been associated with changes in urinary metabolites (Phua et al., 2015) and inflammatory markers (Karl et al., 2017).”

L158-159: Is the DSAT validated? If so, what are the pro and cons of this method?

The DSAT has been validated against isotopes method (Cr- EDTA/^I4^C-mannitol) and delayed-release, methacrylate-coated capsules in healthy non-exercises as a measure of gastrointestinal permeability (Blomquist et al., 1993; Camilleri et al., 2011) and more recently by Ogden and colleagues in healthy exercising men (Ogden et al., 2020). These references were included to illustrate the previous work in the DSAT validation.

Although there is no standardised protocol and the length of collection time for urine are identified as challenges in athletes, urinary appearance of non-digestible saccharides is the most useful test to date to evaluate gastrointestinal cell permeability. These limitations have been highlighted in the end of the review.

L224-225: i-FABP post exercise is not a reliable marker. You need te pre exercise value to get insights in the increase of i-FABP. Please come back to this in the discussion part.

Thank you for highlighting this; we have corrected the sentence as we were referring to the change from baseline rather than just the post-exercise value. The sentence now reads:

**Line 239:** “Morrison et al (2014) showed a larger change from baseline to post-exercise(90mins) in i-FABP levels in trained compared to untrained participants, but showed no effect of colostrum, in spite of the high dosages given (>120g.day^-1^)”

L260-263: What was in the CHO supplement? The effect of glucose for example is different that the effect of an olygosacharide.

Thank you for the comment. The details of each carbohydrate drink are in the overall summary table, but we have included this in the text as well for clarity. It now reads:

**Line 277:** “Of note, Pugh and colleagues included exogenous carbohydrate supplementation during the exercise, due to the length of the exercise bout of ~66g·hr-1 (repeated 22g maltodextrin gels with 200ml water during the running marathon) and ~90g·hr-1 (10% maltodextrin fluid every 15 minutes during 120 minutes of cycling) in the four-strain probiotic studies (Pugh et al., 2019; Pugh et al., 2020).”

L278: there are different kind of measurements taken (blood i-FABP and urinary-i-FABP) which makes the results very questionable.

We agree, but felt that it was premature to exclude the paper. We have highlighted in the discussion that this is not considered a validated measure for endothelial damage. In the discussion, we have added to our previous comment to add clarity:

**Line 532:** “While the study showed a benefit in lower i-FABP levels post 3000m time trial (~9-10 minutes) compared to standard protein supplementation, the study used urinary i-FABP as the marker of endothelial damage adjusted for urinary creatine levels. This may highlight renal clearance of i-FABP rather than systemic appearance, and may require prudent interpretation. This measure has yet to be validated elsewhere and may contribute to the >10 fold increase in concentration.”

L305-311: Rewrite this please. However the risk of bias is low, it has to be undelined that the outcomes are based on specific circumstances.

Thank you for highlighting this, we have added further detail to improve the details around the risk of bias analysis:

**Line 327:** “Overall, the risk of bias across the selected studies was considered low. This was especially clear for sequence generation, allocation concealment and blinding of participants. Four of the twenty-seven studies (15%) used placebo-controlled or matched pairs designs (no crossover), while all other studies used randomised blinded crossover protocols. Due to the nature of the trials, blinding of outcomes was unclear. Five of the studies were registered as clinical trials, meaning that the risk of selective reporting for the remainder was unclear. The risk of bias summary for all studies can be found in Figure 2, and the risk of bias for individual studies are presented in the supplementary data.”

L317-319: Please dig deeper upon the saccharide types.

Thank you for your comment. Unfortunately, the studies that concern mono or disaccharide supplementation are so varied that they allow for very limited comparison. There is no evidence that saccharides will be treated differently at the level of the endothelial past the specific transporters.

L383-384: I would like to see a discourage of usage because of the mentioned reasons.

The authors did not feel it was within our role to make any such statement on this topic because not all athletes are bound by the WADA anti-doping guidelines. We have presented the recent research and have clearly stated the current WADA warnings as objectively as possible, which we believe is appropriate to signpost athlete and allow them to make informed judgements as appropriate.

L409-410: Why is only usage in high temperature highlighted? There are multiple practical implications you could think of.

Our identification of the heated environment is based on the included studies, where all four used heated conditions. While there may be advantages at thermoneutral conditions, none of the studies can confirm this. While there may be other research around glutamine and its use in colonocyte health, these studies are generally not evaluating endothelial dysfunction.

L482-490: What about fiber intake?

Fibre-rich foods would slow down gastric emptying, but may not have impact in relation to monosaccharide levels in the gut. There is no comparable research using food or fibre containing fluids in the acute phase endothelial response to exercise. This will be an interesting avenue to investigate but may contradict the other performance nutrition research and requirements around exercise. We have added a note:

**Line 521:** “Many of the studies target a low FODMAP pre-exercise meal, but there is also no understanding as yet of fibre (in solid or fluid) intake prior to exercise and any impact of gastric emptying on endothelial cell support.”

L614-618: the conclusion is missing the specific environmental settings. Please expend 'context dependent' with some of the factors that may influence GIS.

Thank you for your comment. We have added further details:

**Line 649:** “As such, many of the supplements presented here should be evaluated for their effectiveness in a context dependent manner. External factors such as previous GIS, exercise modality, duration and intra-event fuelling opportunities should contribute to decision making.”

**Reviewer #2:** The authors provide a systematic review of a variety of dietary supplements and their impact on exercise-associated gut damage and gut permeability, primarily measured via Intestinal fatty acid binding protein (i-FABP) and/or dual saccharide absorption tests. The topic is of interested and the review methodology is strong following the PRISMA guidelines. A few suggestions are provided by section below.

Thank you for your time and suggestions to our review.

**Introduction**
Line 86- It would be nice to have more clarity regarding specifically, which gastrointestinal symptoms are associated with increased endotoxin levels, as well as, an estimated time to on-set and duration of the effects.

**Line 86:** Endotoxin levels have been associated with nausea in ultra-endurance athletes (Stuempfle et al., 2016) but not in shorter duration exercise (Moncada-Jiménez et al., 2009). As a consequence, the local and systemic responses may contribute to higher rates of gastrointestinal symptoms (Stuempfle et al., 2016).

Line 88 – Changes in gastric emptying and motility may affect nutrient absorption. Consider discussing, as this may affect gut permeability and be relevant to the saccharide absorption tests.

Thank you for highlighting this. We have added a sentence as such to expand on this concept within the research

**Line 91:** “Gastroparesis or ‘slosh stomach’ may contribute to reduced nutrient absorption or gastrointestinal cell injury, but the overall rates and consequences in athletes are unknown (Biondich & Joslin, 2016).”

Line 99 – Given that some of the studies use an acute supplementation and chronic, it would be valuable to discuss potential mechanism and/ore effects of both. For instance how would an chronic supplementation impact gut permeability during a bout of exercise vs. an acute protocol.

This is an interesting comment and we have added a sentence to add to the introduction.

Line 104: “Further, the impact of chronic or acute supplementation protocols with similar supplements has not been considered.”

**Methods**

Why were your outcome measures not included in your “key word” search list?

Thank you for your comment. Due to the original scope of the review (to be as wide as possible), we made allowances for other markers to be used. This included fecal zonulin, fecal Calprotectin, or Iohexol. However, in the review process, we discovered that the DSAT and i-FABP tests were the most appropriate options to approximate endothelial damage and permeability, and that other markers were not used in athlete populations.

Line 138 – It would help the reader to clarify that the ratio of lactulose to rhamnose/ mannose is used and that an increase in lactulose indicates increased permeability.

Thank you for highlighting. We have added some further explanation. It now reads:

**Line 144** “Lactulose (disaccharide) absorption and appearance in the urine/plasma will increase with disturbances in endothelial gap junctions while mannitol/rhamnose (monosaccharide) absorption is used as a normalising factor for lactulose since it utilises paracellular transport. An increase in the ratio of the larger disaccharide to the smaller monosaccharide illustrates and increase in paracellular passage and hence intestinal permeability.”

Line 164 – inclusion criteria for the exercise would be valuable if there were any. i.e. endurance exercise etc. It appears there are no studies looking at strength or intermittent exercise. Note if they were specifically excluded or simply not available. I see there is some discussion on this in the limitations with respect to “team” sports.

This is a good observation since there are currently no studies evaluating these markers in team sports or resistance exercise. The single study in resistance exercise (Wijck, van et al., 2013) was excluded due to the lack of control group with regards to the whey protein intake. The study showed an increase in permeability and endothelial damage presented as part of the meta-analysis by the authors (Chantler et al., 2020). This is a large gap in the current research and one aspect of our future research.

Finally, were there any criteria regarding the amount of exercise pre-test day i.e. after a “rest day” or dietary controls i.e. “no caffeine” or drug controls i.e. “no NSAIDS”?

While this is standardised amongst the research studies, we have included for clarity:

**Line 150:** “To improve the robustness of these markers, participants are asked to avoid alcohol, spicy food, non-steroidal anti-inflammatories, caffeine or high intensity exercise in the day preceding exercise trials (Costa et al., 2017; Ogden et al., 2020).”

**Results**
Table 1 is comprehensive and well done. Table description for clarity confirm that difference is significant and p-value used.

Thank you, we have added this to the legend of the table.

Table 1 – last row/column should “an” be “and”?

Thank you for spotting this – it has been corrected

Line 281 - Macronutrients can any statements be made regarding liquid vs solid based on the data?

The exercise and carbohydrate protocols are diverse and too limited in number to be able to make any comment with reasonable assertion. We have added more detail to this paragraph to clarify:

**Line 287:** “Four studies using carbohydrate fluids (of various concentrations; 6-16% solution) mitigated the impact of exercise on i-FABP concentrations (Flood et al., 2020; Jonvik et al., 2019; Lambert et al., 2008; Snipe et al., 2017) although all used different saccharide combinations and concentrations limiting comparison (specific values in Table 1).”

**Discussion**
Bovine Colostrum – some insight into potential mechanism would be of interest if available. The in vitro studies do mention potential protective measures, but how these could be occurring is not discussed.

Thank you for your comment. We have briefly mentioned some of the mechanistic data (Line 354) and due to the overall length of the review felt it was important to limit the length of these explanations.

We have added one additional sentence to reference further reviews of the research in this area:

**Line 362:** “Some of the mechanisms have been investigated and reviewed (58) but will require further research around exercise to elucidate further.”

Line 356 – “affect” should be “effect”

It is odd that in line 305 it was noted that subgroup analysis could not occur and yet later it is noted that training status did not alter the response (line 368). I’m not sure there is sufficient data here to support the statement.

Thank you for the comment. The comparison of trained and untrained groups was derived from the study itself, not from any subgroup analysis by the authors. It is highlighted later in the paragraph that this is the only study to consider this question so far, and that further research would be useful to elucidate details around the role of fitness or training and gastrointestinal splanchnic response and consequent GI damage and permeability. We have adjusted the paragraph for clarity:

**Line 390:** “In the single study that considered trained and untrained participants, training status did not alter the response to bovine colostrum supplementation compared to the placebo condition. The authors did note a higher i-FABP concentration post-exercise in the trained participants compared to untrained, but this was independent of colostrum supplementation (Morrison et al., 2014). Higher output during exercise based on training status reflected in body temperature could have contributed to this difference, but there was no significant difference in the body temperature during exercise between groups.”

Line 383 “it’s” should be “its”

Thank you, this has been changed.

Consider expanding the discussion on heat shock protein and occludin to relate back to the current studies. One or two sentences to explain to the reader how these affect gut permeability should be sufficient.

Thank you for your comment. We have added an additional sentence to link the mechanisms to the possible benefits.

**Line 435:** “Heat shock protein upregulation can improve cell tolerance to oxidative stress and inflammation and interact with occludin to preserve tight junction integrity (Arnal & Lallés, 2016). This is reflected in the lower permeability markers (L:R) levels seen by Zuhl and colleagues (Zuhl, M. et al., 2015; Zuhl, M. N. et al., 2014).”

Line 574 – “marker” should be “markers”

Thank you for spotting this error. It has been changed

**Limitations**
Potentially lack of controls in the study regarding caffeine and NSAIDs as mentioned in methods sections.

This has been added to the methods to clarify as it is generally standard practice in the included studies.

**Line 150: “**To improve the robustness of these markers, it is standard practice for participants to avoid alcohol, spicy food, non-steroidal anti-inflammatories, caffeine or high intensity exercise in the day preceding exercise trials (Costa et al., 2017; Ogden et al., 2020).”

Line 597 – I also didn’t see studies in strength based or HITT/cross-fit type workouts

Please see our previous comment – since there are no studies to date to the authors knowledge.

Line 601 – females generally experience higher levels of exercise-induced GI symptoms than males.

While this is true in the earlier studies, the recent review by Pugh et al states that there may be confounding factors such as smaller representation in endurance events and lower training age for women compared to men (Pugh et al., 2021). However, this is still worthwhile investigating further and is part of our future research.

**References:**

Arnal, M. E. & Lallés, J. P. (2016) Gut Epithelial Inducible Heat-Shock Proteins and Their Modulation by Diet and the Microbiota. *Nutrition Reviews*, 74 (3), pp. 181–197.

Biondich, A. S. & Joslin, J. D. (2016) Quantifying the “Slosh Stomach”: A Novel Tool for Assessment of Exercise-Associated Gastroparesis Symptoms in Endurance Athletes. *Journal of Sports Medicine*, 2016, pp. 1–5.

Blomquist, L., Bark, T., Hedenborg, G., Svenberg, T. & Norman, A. (1993) Comparison between the Lactulose/Mannitol and 51cr-Ethylenediaminetetraacetic Acid/14c-Mannitol Methods for Intestinal Permeability Frequency Distribution Pattern and Variability of Markers and Marker Ratios in Healthy Subjects. *Scandinavian Journal of Gastroenterology*, 28 (3), pp. 274–280.

Camilleri, M., Nadeau, A., Lamsam, J., Linker Nord, S., Ryks, M., Burton, D., Sweetser, S., Zinsmeister, A. R. & Singh, R. J. (2011) Understanding Measurements of Intestinal Permeability in Healthy Humans with Urine Lactulose and Mannitol Excretion. *Neurogastroenterol Motil*, 22 (1), pp. 1–22.

Chantler, S., Griffiths, A., Matu, J., Davison, G., Jones, B. & Deighton, K. (2020) The Effects of Exercise on Indirect Markers of Gut Damage and Permeability: A Systematic Review and Meta-Analysis. *Sports Medicine*.

Costa, R. J. S., Snipe, R. M. J., Kitic, C. M. & Gibson, P. R. (2017) Systematic Review : Exercise-Induced Gastrointestinal Syndrome — Implications for Health and Intestinal Disease. *Alimentary pharmacology & therapeutics*, (March), pp. 246–265.

Davison, G., Marchbank, T., March, D. S., Thatcher, R. & Playford, R. J. (2016) Zinc Carnosine Works with Bovine Colostrum in Truncating Heavy Exercise-Induced Increase in Gut Permeability in Healthy Volunteers. *American Journal of Clinical Nutrition*, 104 (2) August, pp. 526–536.

Dokladny, K., Zuhl, M. N. & Moseley, P. L. (2016) Intestinal Epithelial Barrier Function and Tight Junction Proteins with Heat and Exercise. *Journal of Applied Physiology*, 120 (6) March, pp. 692–701.

Flood, T. R., Montanari, S., Wicks, M., Blanchard, J., Sharpe, H., Taylor, L., Kuennen, M. R. & Lee, B. J. (2020) Addition of Pectin-Alginate to a Carbohydrate Beverage Does Not Maintain Gastrointestinal Barrier Function during Exercise in Hot-Humid Conditions Better than Carbohydrate Ingestion Alone. *Applied Physiology, Nutrition, and Metabolism*, pp. 1–37.

Horowitz, M., Cunningham, K. M., Wishart, J. M., Jones, K. L. & Read, N. W. (1996) The Effect of Short-Term Dietary Supplementation with Glucose on Gastric Emptying of Glucose and Fructose and Oral Glucose Tolerance in Normal Subjects. *Diabetologia*, 39 (4), pp. 481–486.

Jonvik, K. L., Lenaerts, K., Smeets, J. S. J., Kolkman, J. J., Loon, L. J. C. Van & Verdijk, L. B. (2019) Sucrose but Not Nitrate Ingestion Reduces Strenuous Cycling-Induced Intestinal Injury. *Medicine and Science in Sports and Exercise*, 51 (3), pp. 436–444.

Karl, J. P., Margolis, L. M., Madslien, E. H., Murphy, N. E., Castellani, J. W., Gundersen, Y., Hoke, A. V., Levangie, M. W., Kumar, R., Chakraborty, N., Gautam, A., Hammamieh, R., Martini, S., Montain, S. J. & Pasiakos, S. M. (2017) Changes in Intestinal Microbiota Composition and Metabolism Coincide with Increased Intestinal Permeability in Young Adults under Prolonged Physiological Stress. *American Journal of Physiology - Gastrointestinal and Liver Physiology*, 312 (6) June, pp. G559–G571.

Lambert, G. P., Lang, J., Bull, A., Pfeifer, P. C., Eckerson, J., Moore, C., Lanspa, S. & O’Brien, J. (2008) Fluid Restriction during Running Increases GI Permeability. *International Journal of Sports Medicine*, 29 (3) March, pp. 194–198.

Moncada-Jiménez, J., Plaisance, E., Mestek, M. L., Araya-Ramírez, F., Ratcliff, L., Taylor, J. K., Grandjean, P. W. & AragónVargas, L. F. (2009) Initial Metabolic State and Exercise-Induced Endotoxaemia Are Unrelated to Gastrointestinal Symptoms during Exercise. *Journal of Sports Science and Medicine*, 8 (2) June, pp. 252–258.

Morrison, S. A., Cheung, S. S. & Cotter, J. D. (2014) Bovine Colostrum, Training Status, and Gastrointestinal Permeability during Exercise in the Heat: A Placebo-Controlled Double-Blind Study. *Applied Physiology, Nutrition and Metabolism*, 39 (9), pp. 1070–1082.

Ogden, H. B., Fallowfield, J. L., Child, R. B., Davison, G., Fleming, S. C., Edinburgh, R. M., Delves, S. K., Millyard, A., Westwood, C. S. & Layden, J. D. (2020) Reliability of Gastrointestinal Barrier Integrity and Microbial Translocation Biomarkers at Rest and Following Exertional Heat Stress. *Physiological Reports*, 8 (5), pp. 1–16.

Oliveira, E. P. de & Burini, R. C. (2014) Carbohydrate-Dependent, Exercise-Induced Gastrointestinal Distress. *Nutrients*, 6 (10), pp. 4191–4199.

Phua, L. C., Wilder-Smith, C. H., Tan, Y. M., Gopalakrishnan, T., Wong, R. K., Li, X., Kan, M. E., Lu, J., Keshavarzian, A. & Chan, E. C. Y. (2015) Gastrointestinal Symptoms and Altered Intestinal Permeability Induced by Combat Training Are Associated with Distinct Metabotypic Changes. *Journal of Proteome Research*, 14 (11) November, pp. 4734–4742.

Pugh, J. N., Lydon, K., O’Donovan, C. M., O’Sullivan, O. & Madigan, S. M. (2021) More than a Gut Feeling: What Is the Role of the Gastrointestinal Tract in Female Athlete Health? *European Journal of Sport Science*, pp. 1–10.

Pugh, J. N., Sparks, A. S., Doran, D. A., Fleming, S. C., Langan-Evans, C., Kirk, B., Fearn, R., Morton, J. P. & Close, G. L. (2019) Four Weeks of Probiotic Supplementation Reduces GI Symptoms during a Marathon Race. *European Journal of Applied Physiology*, 119 (7), pp. 1491–1501.

Pugh, J. N., Wagenmakers, A. J. M., Doran, D. A., Fleming, S. C., Fielding, B. A., Morton, J. P. & Close, G. L. (2020) Probiotic Supplementation Increases Carbohydrate Metabolism in Trained Male Cyclists: A Randomized, Double-Blind, Placebo-Controlled Crossover Trial. *American Journal of Physiology - Endocrinology and Metabolism*, 318 (4), pp. E504–E513.

Sessions, J., Bourbeau, K., Rosinski, M., Szczygiel, T., Nelson, R., Sharma, N. & Zuhl, M. (2016) Carbohydrate Gel Ingestion during Running in the Heat on Markers of Gastrointestinal Distress. *European Journal of Sport Science*, 16 (8) November, pp. 1064–1072.

Snipe, R. M. J., Khoo, A., Kitic, C. M., Gibson, P. R. & Costa, R. J. S. (2017) Carbohydrate and Protein Intake during Exertional-Heat Stress Ameliorates Intestinal Epithelial Injury and Small Intestine Permeability. *Appl. Physiol. Nutr. Metab*, 42 (November) August, pp. 1–41.

Stuempfle, K. J., Valentino, T., Hew-Butler, T., Hecht, F. M. & Hoffman, M. D. (2016) Nausea Is Associated with Endotoxemia during a 161-Km Ultramarathon. *Journal of Sports Sciences*, 34 (17), pp. 1662–1668.

Wijck, K. van, Lenaerts, K., Grootjans, J., Wijnands, K. A. P., Poeze, M., Loon, L. J. C. van, Dejong, C. H. C. & Buurman, W. A. (2012) Physiology and Pathophysiology of Splanchnic Hypoperfusion and Intestinal Injury during Exercise: Strategies for Evaluation and Prevention. *American Journal of Physiology - Gastrointestinal and Liver Physiology*, 303 (2) July, pp. G155–G168.

Wijck, K. van, Lenaerts, K., Loon, L. J. C. van, Peters, W. H. M., Buurman, W. A. & Dejong, C. H. C. (2011) Exercise-Induced Splanchnic Hypoperfusion Results in Gut Dysfunction in Healthy Men. *PLoS ONE*, 6 (7) July, p. e22366.

Wijck, K. van, Pennings, B., Bijnen, A. A. van, Senden, J. M. G. G., Buurman, W. A., Dejong, C. H. C. C., Loon, L. J. C. van, Lenaerts, K., Wijck, K. Van, Pennings, B., Bijnen, A. A. Van, Senden, J. M. G. G., Buurman, W. A., Dejong, C. H. C. C., Loon, L. J. C. Van, Lenaerts, K., Wijck, K. van, Pennings, B., Bijnen, A. A. van, Senden, J. M. G. G., Buurman, W. A., Dejong, C. H. C. C., Loon, L. J. C. van & Lenaerts, K. (2013) Dietary Protein Digestion and Absorption Are Impaired during Acute Postexercise Recovery in Young Men. *American Journal of Physiology - Regulatory Integrative and Comparative Physiology*, 304 (5), pp. 356–361.

Wilson, P. B. & Ingraham, S. J. (2015) Glucose-Fructose Likely Improves Gastrointestinal Comfort and Endurance Running Performance Relative to Glucose-Only. *Scandinavian Journal of Medicine and Science in Sports*, 25 (6), pp. e613–e620.

Zuhl, M., Dokladny, K., Mermier, C., Schneider, S., Salgado, R. & Moseley, P. (2015) The Effects of Acute Oral Glutamine Supplementation on Exercise-Induced Gastrointestinal Permeability and Heat Shock Protein Expression in Peripheral Blood Mononuclear Cells. *Cell Stress and Chaperones*, 20 (1), pp. 85–93.

Zuhl, M. N., Lanphere, K. R., Kravitz, L., Mermier, C. M., Schneider, S., Dokladny, K. & Moseley, P. L. (2014) Effects of Oral Glutamine Supplementation on Exercise-Induced Gastrointestinal Permeability and Tight Junction Protein Expression. *Journal of Applied Physiology*, 116 (2) January, pp. 183–191.
